# Two cases of intrauterine pregnancy with tubal stump pregnancy after in vitro fertilization and embryo transfer following ipsilateral salpingectomy: A case report

**DOI:** 10.1097/MD.0000000000018183

**Published:** 2019-12-10

**Authors:** Qi Xi, Yang Yu, Xinyue Zhang, Hongguo Zhang, Yuting Jiang, Ruizhi Liu, Han Zhang

**Affiliations:** Center for Reproductive Medicine, Center for Prenatal Diagnosis, The First Hospital, Jilin University, Changchun, Jilin, P.R. China.

**Keywords:** heterotopic pregnancy, in vitro fertilization and embryo transfer, salpingectomy, tubal stump pregnancy

## Abstract

**Rationale:**

The recently increased rate of heterotopic pregnancies (HPs) has been largely attributed to the increased use of assisted reproduction technologies (ARTs). HP is a rare and potentially life-threatening condition. It is unusual in natural conception cycles, occurring in 1:10,000 to 1:50,000 pregnancies. However, with the increased use of ART such as in vitro fertilization and embryo transfer (IVF–ET), the incidence has risen to 0.5–1%.

**Patient concerns:**

Case 1 was a 28-year-old woman who presented to our center complaining of a sudden onset of right-side lower abdominal pain with a small amount of vaginal bleeding. She had undergone IVF–ET and received a thawed embryo transfer with two embryos 23 days previously. She had a history of right salpingectomy for an ectopic pregnancy during the downregulation of her ovulatory cycle 1 year ago. Case 2 was a 25-year-old woman who presented to our center complaining of a sudden onset of right-side lower abdominal pain. She had also undergone thawed embryo transfer following IVF–ET with two embryos 35 days previously. She had a history of right salpingectomy for an ectopic pregnancy 1.5 years previously.

**Diagnoses:**

Both patients were diagnosed as having a heterotopic pregnancy.

**Interventions:**

Patient 1 underwent emergency laparoscopy; patient 2 underwent emergency laparotomy and both were treated medically to prevent abortion of the intrauterine pregnancies.

**Outcomes:**

Patient 1 had an incomplete abortion and underwent uterine curettage on the day 10 after the operation. Patient 2 experienced no further complications during pregnancy and a healthy baby girl was born at the 38th gestational week.

**Lessons:**

Reproductive physicians need to pay more attention to patients who have received more than one embryo at transfer, especially those with a history of salpingectomy.

## 1 Introduction

Heterotopic pregnancy (HP) is a rare and potentially life-threatening condition. It is unusual in natural conception cycles, occurring in 1:10,000 to 1:50,000 pregnancies.^[1]^ However, the incidence is rising to 0.5–1% with the increased use of assisted reproduction technologies (ARTs).^[2,3]^ Moreover, tubal stump pregnancies account for 1.16% of all ectopic pregnancies.^[4]^ In vitro fertilization and embryo transfer (IVF–ET) is known to increase the incidence of HP, but there are few reports about intrauterine combined with tubal stump pregnancies following such procedures. We report two cases of intrauterine pregnancy combined with tubal stump pregnancy following IVF–ET with the aim of alerting reproductive physicians to pay more attention to the possibility of this type of HP.

## 2 Case presentations

Approval by an ethics committee or institutional review board was not necessary, because this was a noninvasive follow-up observational study. We obtained verbal informed consent from the patients to report this study.

### 2.1 Case 1

**Figure 1:**
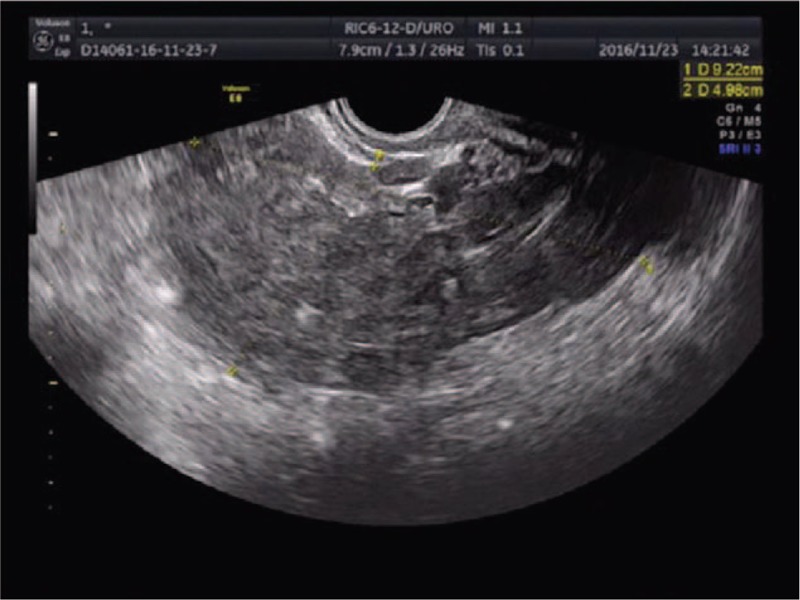
An uneven mass about 9.2 × 5.0 cm at the right adnexal.

**Figure 2:**
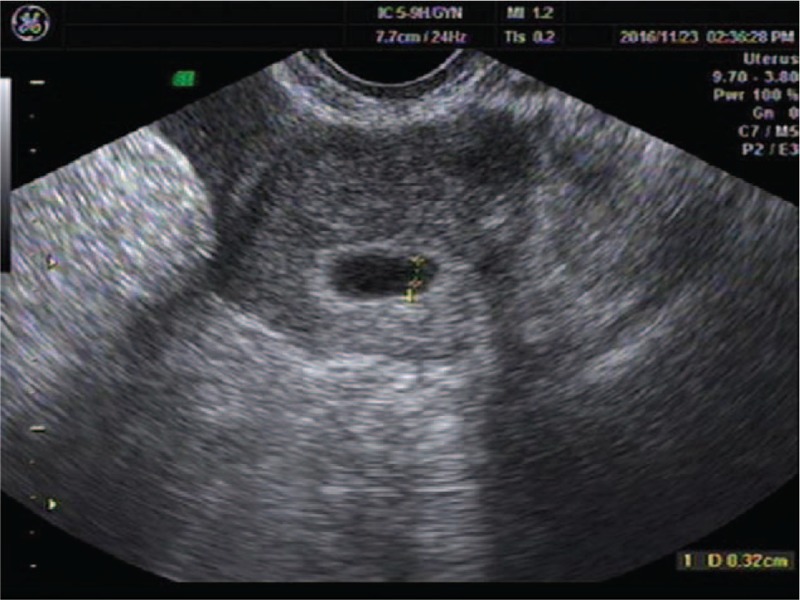
An intrauterine gestational sac about 1.0 × 1.1 × 0.8 cm with just a yolk sac about 0.3 cm.

**Figure 3:**
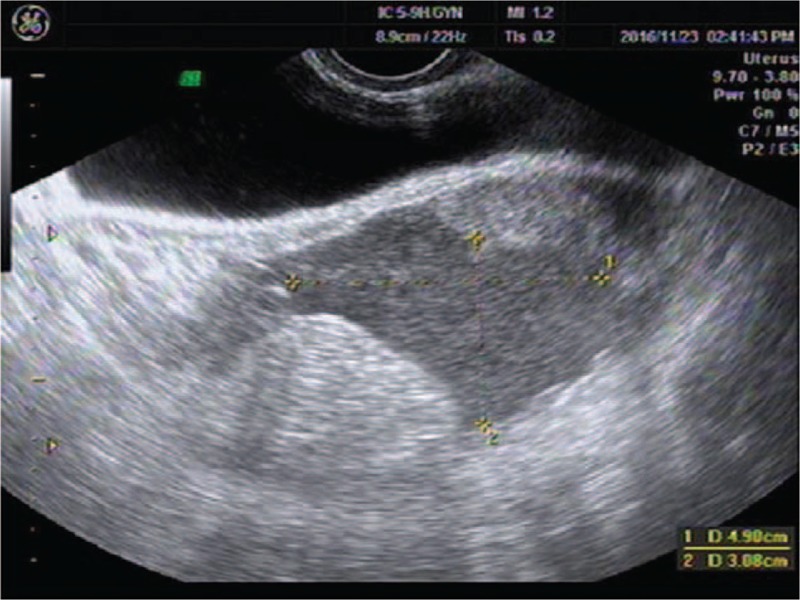
Ultrasonographic evidence of hemoperitoneum was present with a large amount of free fluid within the pouch of Douglas.

**Figure 4:**
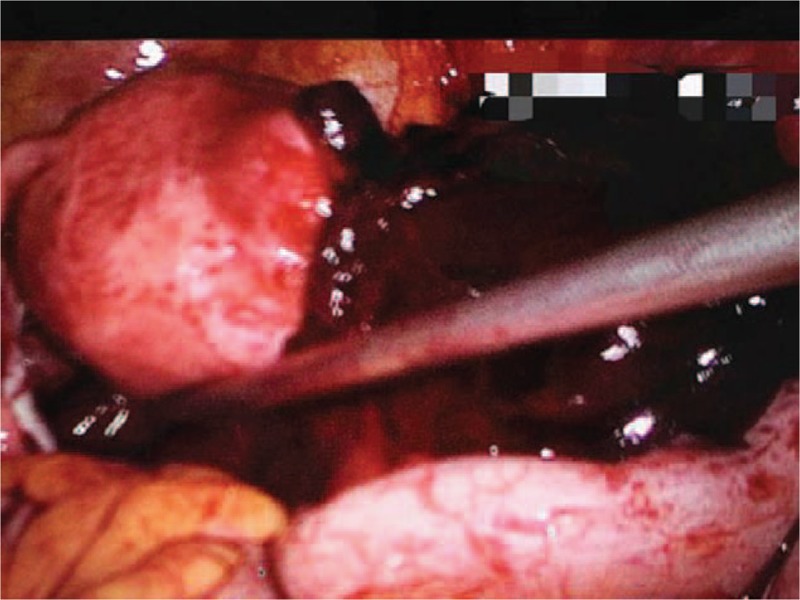
A ruptured right interstitial pregnancy hemoperitoneum.

A 28-year-old woman presented to our center complaining of a sudden onset of right-side lower abdominal pain with slight vaginal bleeding. She had undergone a thawed ET with two embryos 23 days before, following IVF. She had a history of right salpingectomy for an ectopic pregnancy during ovulatory cycle downregulation for IVF–ET 1 year previously. On physical examination, she was hemodynamically stable with a blood pressure of 97/65 mm Hg and heart rate of 80 bpm. A physical examination demonstrated a distended abdomen with diffuse abdominal pain that was maximal in the right iliac fossa with rebound pain and signs of peritonitis. Transvaginal sonography demonstrated one intrauterine gestational sac with an uneven mass measuring about 9.2 × 5.0 cm at the right adnexa (Fig. 1). The intrauterine gestational sac measured 1.0 × 1.1 × 0.8 cm with a yolk sac about 0.3 cm in diameter (Fig. 2). Ultrasonographic evidence of hemoperitoneum was present, with a large amount of free fluid within the pouch of Douglas (Fig. 3). The patient underwent emergency laparoscopy that confirmed a ruptured right interstitial pregnancy and hemoperitoneum (Fig. 4). This ectopic pregnancy tissue was removed and effective hemostasis achieved. The amount of hemoperitoneum was approximately 1.0 L, which was evacuated followed by peritoneal lavage. Histopathology of the tissues confirmed a tubal stump pregnancy. Medical treatment of progesterone and estrogen was used to prevent abortion of the intrauterine pregnancy. However, on day 10 after the operation, the patient had an incomplete abortion and underwent uterine curettage.

### 2.2 Case 2

**Figure 5:**
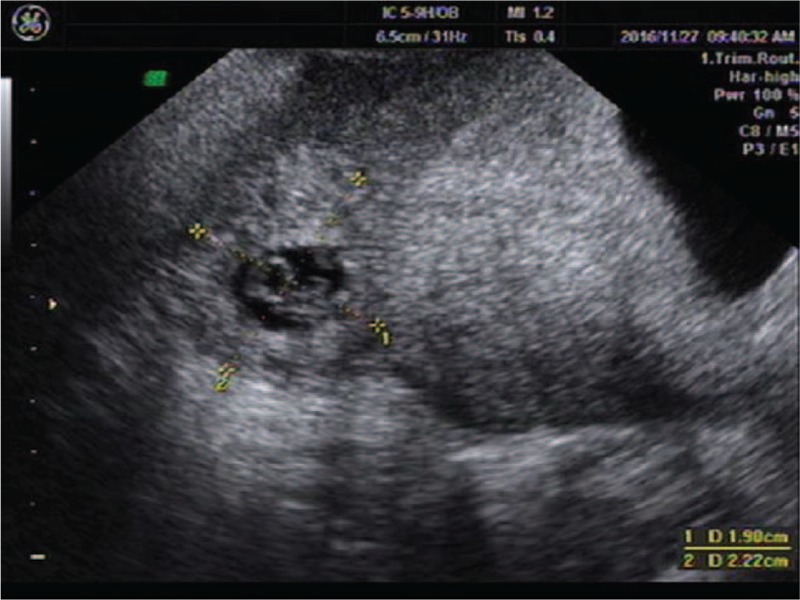
An intrauterine gestational sac and an uneven mass about 2.2 × 2.0 cm at the right adnexal.

**Figure 6:**
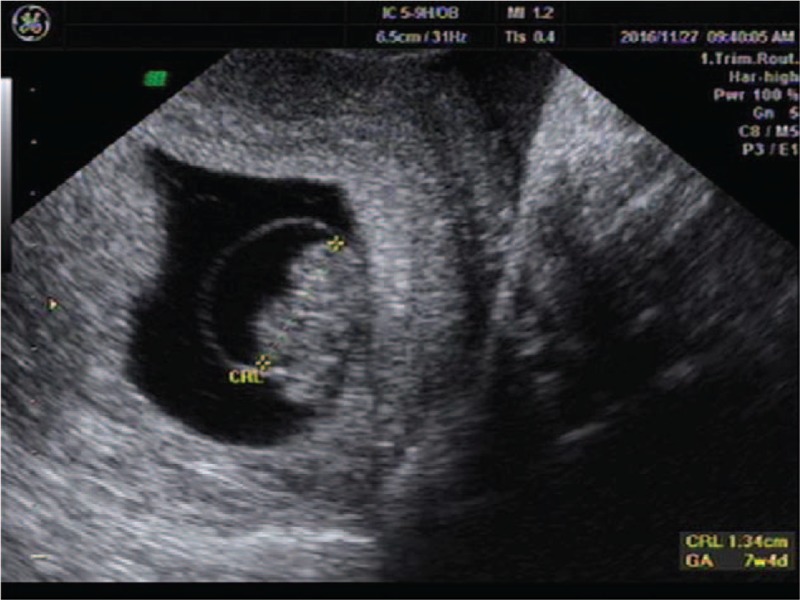
A crown rump length of the intrauterine fetus about 1.4 cm corresponding to gestation of 7 weeks.

**Figure 7:**
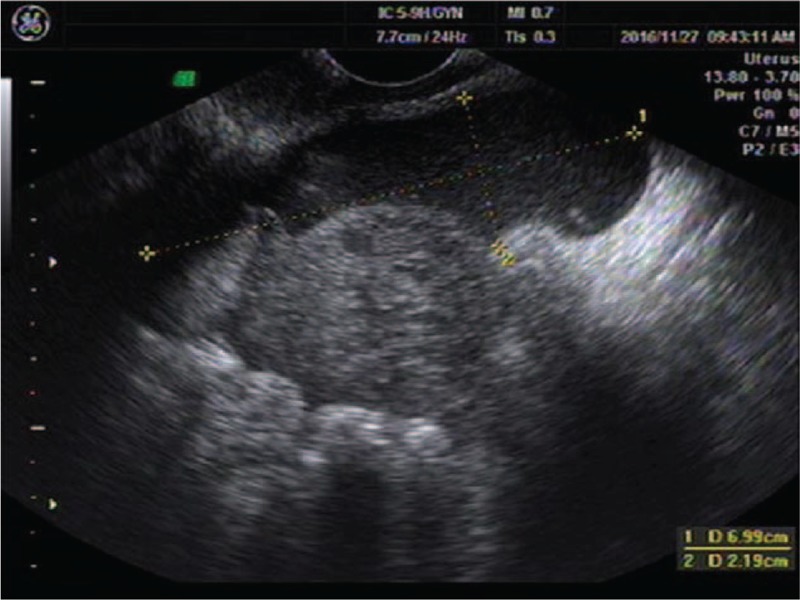
Ultrasonographic evidence of hemoperitoneum was present with a large amount of free fluid within the pouch of Douglas.

A 25-year-old woman presented to our center complaining of a sudden onset of right-side lower abdominal pain. She had undergone a thawed ET with two embryos 35 days ago following IVF. She had a history of right salpingectomy for an ectopic pregnancy 1.5 years ago. On physical examination, she was hemodynamically stable with a blood pressure of 110/79 mm Hg and heart rate of 98 bpm. A physical examination demonstrated a distended abdomen with diffuse abdominal tenderness that was maximal in the right iliac fossa with rebound tenderness and signs of peritonitis. Her serum beta-human chorionic gonadotropin (β-hCG) concentration was 63,212 mIU/mL. Transvaginal ultrasonography demonstrated one intrauterine gestational sac, with an uneven mass measuring about 2.2 × 2.0 cm at the right adnexa (Fig. 5). The crown-rump length of the intrauterine fetus was 1.4 cm, corresponding to a gestation length of 7 weeks (Fig. 6). Ultrasonographic evidence of hemoperitoneum was present, with a large amount of free fluid within the pouch of Douglas (Fig. 7).

The patient underwent emergency laparotomy that confirmed a ruptured right tubal stump pregnancy with hemoperitoneum. The ectopic tissue was removed and effective hemostasis achieved. The volume of hemoperitoneum was approximately 0.3 L, which was evacuated followed by peritoneal lavage. Histopathology of the tissues confirmed a tubal stump pregnancy. Medical treatment of progesterone and estrogen was used to prevent abortion of the intrauterine pregnancy. Regular prenatal examination was performed with no further complications during the subsequent pregnancy. A healthy baby girl was born at 38 gestational weeks.

## 3 Discussion and conclusions

It is considered that the risk of repeated ectopic pregnancies in the Fallopian tubes can be eliminated by salpingectomy.^[5]^ The exact mechanism of tubal stump pregnancy is elusive. One possible explanation is that an embryo in one Fallopian tube is carried to the contralateral remnant tube by intrauterine fluids. The appearance of a corpus luteum in the contralateral ovary has been reported and might support this speculation.^[6]^ However, prevention of this type of ectopic pregnancy has not been reported. Theoretically, if complete resection of the tube is performed during the initial salpingectomy, the occurrence of an ectopic pregnancy could be prevented in the isthmic portion of the remnant tube. However, spontaneous occurrence of corneal/interstitial pregnancies after ipsilateral salpingectomy has been reported.^[4,7]^

HP following salpingectomy is extremely rare. Xu et al reported an HP after bilateral salpingectomy with subsequent intrauterine and ovarian implantations. They suggested that this might have involved the migration of one of the embryos through a microscopic fistulous tract in the interstitial portion of the tube and its subsequent implantation in the ovary.^[8]^ It was suggested that the volume of culture fluid and the increased pressure of the implanted embryo or the downward tilt of the patient during ET might be one cause of ectopic pregnancy.^[9]^ Here, we describe two patients who had an intrauterine pregnancy combined with a tubal stump pregnancy after IVF–ET following previous ipsilateral salpingectomy. To our knowledge there have been just two such cases in our department since May 2011.

The diagnosis of HP is difficult. Most cases have been discovered because of sudden abdominal pain following rupture of the ectopic pregnancy. Transvaginal ultrasonography is the most important diagnostic method. Thus, examination has been suggested to be performed at regular intervals especially when multiple embryos have been transferred following IVF–ET.^[8]^ Kasum et al suggested that HP should always be suspected in the differential diagnosis of symptomatic patients with an intrauterine pregnancy following IVF–ET.^[10]^ Shavit et al suggested that the possibility of a HP should be considered when more than one embryo has been transferred in an IVF–ET cycle.^[11]^ In this case report, two patients presented with lower abdominal pain after ET. Patient 1 showed lower abdominal pain 23 days after ET, and ultrasonography indicated an intrauterine pregnancy combined with an ectopic tubal stump pregnancy. Therefore, a history of tubal resection among patients undergoing IVF–ET suggests that if there is a small amount of vaginal bleeding or lower abdominal pain, it is necessary to consider the possibility of a tubal stump pregnancy and patients should be advised to return to the hospital for transvaginal ultrasonography.

The principal treatment of a tubal stump pregnancy is surgery. Because the implantation sites are prone to rupture associated with abdominal bleeding, this is a life-threatening situation and needs emergency surgery. The first choice is laparoscopic surgery with complete resection of the Fallopian tube stump as the basic treatment. This is because such pregnancies are generally in the tubal interstitium at the uterotubal junction where the muscular layer is thicker with a rich blood supply and more bleeding in the operation. Studies have shown that abdominal pain or vaginal bleeding before diagnosis are predictors of miscarriage.^[12,13]^ Another study showed that gestational age at treatment was the only independent risk factor for miscarriage in patients with an HP.^[14]^ With increased awareness of the possibility of HP among patients who have undergone IVF–ET, early diagnosis and appropriate surgical treatment may lead to a favorable prognosis, regardless of the method for treatment of the ectopic pregnancy.^[15,16]^ Here, the ectopic pregnancy in Case 1 ruptured at an early stage as the intrauterine gestational sac measured only 1.0 × 1.1 × 0.8 cm. Miscarriage could not be avoided after the surgery. Case 2 was discovered before rupture of the ectopic pregnancy lesion followed by a successful term pregnancy. Therefore, early diagnosis of HP could help salvage the birth outcome in such cases.

Reproductive physicians need to pay more attention to patients who have received more than one embryo in IVF–ET cycles, even though intrauterine pregnancies combined with tubal stump pregnancies following ipsilateral salpingectomy are rare.

## Acknowledgments

We are grateful to the patients, who gave their informed consent for publication.

## Author contributions

**Conceptualization:** Qi Xi.

**Data curation:** Qi Xi, Yang Yu.

**Investigation:** Xinyue Zhang, Hongguo Zhang.

**Resources:** Yuting Jiang.

**Supervision:** Han Zhang.

**Writing – original draft:** Qi Xi.

**Writing – review & editing:** Qi Xi, Ruizhi Liu, Han Zhang.

Han Zhang orcid: 0000-0003-4469-3809.
